# Complete Genome Sequence of the Acetogenic Bacterium *Moorella thermoacetica* DSM 2955^T^

**DOI:** 10.1128/genomeA.01157-15

**Published:** 2015-10-08

**Authors:** Frank R. Bengelsdorf, Anja Poehlein, Carola Esser, Bettina Schiel-Bengelsdorf, Rolf Daniel, Peter Dürre

**Affiliations:** a Institut für Mikrobiologie und Biotechnologie, Universität Ulm, Ulm, Germany; b Genomic and Applied Microbiology & Göttingen Genomics Laboratory, Georg-August University, Göttingen, Germany

## Abstract

Here, we report the complete genome sequence of *Moorella thermoacetica* DSM 2955^T^, an acetogenic bacterium, which uses the Wood–Ljungdahl pathway for reduction of H_2_ + CO_2_ or CO. The genome consists of a single circular chromosome (2.62 Mb).

## GENOME ANNOUNCEMENT

We determined the genome sequence of one of the type strains of *Moorella thermoacetica* DSM 2955^T^, originally named *Clostridium thermoaceticum*, which was isolated from horse feces in 1942 (1). About 40 years later, Kerby and Zeikus (2) obtained the original isolated strain from Elizabeth McCoy as a “dried spore stock in soil” and showed that this strain uses H_2_ + CO_2_ as its sole carbon and energy source. Moreover, this strain was adapted to use CO as its sole substrate (2). These were major differences from the previously submitted type strain (DSM 521^T^), and therefore *M. thermoacetica* DSM 2955^T^ was deposited in “Deutsche Sammlung von Mikroorganismen und Zellkulturen GmbH” (DSMZ) as the second type strain of the species. However, since isolation of *M. thermoacetica*, the strains DSM 521^T^ and ATCC 39073 were used as model acetogens for elucidating the biochemistry of the Wood-Ljungdahl pathway (3, 4). In 2008, Pierce et al. (5) published the first complete genome sequence of the non-type strain *M. thermoacetica* ATCC 39073.

Chromosomal DNA was isolated using a MasterPure complete DNA purification kit (Epicentre, Madison, WI, USA) and used to create Illumina shotgun paired-end sequencing libraries. A MiSeq system and MiSeq reagent kit v3 (600 cycles) were used for sequencing as recommended by the manufacturer (Illumina, San Diego, CA, USA). Sequencing resulted in 2,908,542 paired-end reads (2 × 300 bp). Trimmomatic 0.32 (6) was used for quality filtering and adaptor clipping. The *de novo* assembly was performed by employing the SPAdes genome assembler software 3.5.0 (7), which resulted in 43 contigs (>500 bp) with a 231-fold average coverage. For scaffolding, the Move Contigs tool of the Mauve genome alignment software (8) was used together with the genome sequence of *M. thermoacetica* ATCC 39073 (CP000232) as reference. Remaining gaps were closed by PCR-based techniques, primer walking, and Sanger sequencing of the products using BigDye 3.0 chemistry and an ABI3730XL capillary sequencer (Life Technologies GmbH, Darmstadt, Germany). For this purpose, the Gap4 (v4.11) software of the Staden package (9) was employed.

The genome of *M. thermoacetica* DSM 2955^T^ consists of a circular chromosome of 2.62 Mb with an overall G+C content of 55.81%. Automatic gene prediction was performed by using Prodigal (10). Genes coding for rRNA and tRNA were identified using RNAmmer (11) and tRNAscan (12), respectively. The Integrated Microbial Genomes–Expert Review (IMG-ER) system (13) was used for automatic annotation, which was subsequently manually curated by using the Swiss-Prot, TrEMBL, and InterPro databases (14). The genome harbored 3 rRNA genes, 52 tRNA genes, and 2,624 protein-encoding genes. The genome of strain DSM 2955^T^ (2,623,349 bp) is smaller than that of strain ATCC 39073 (2,628,784 bp), but larger than that of the recently published strain DSM 521^T^ (2,527,564 bp) (15). It harbors additional genes encoding, e.g., hypothetical proteins, methylases, and transposases. Genome comparisons of strain DSM 2955^T^ with strain ATCC 39073 and strain DSM 2955^T^ with strain DSM 521^T^ revealed 120 single nucleotide polymorphisms (SNPs) and 936 SNPs, respectively.

### Nucleotide sequence accession number.

This complete genome project has been deposited at DDBJ/EMBL/GenBank under the accession number CP012370.
